# Epidemiologic study of accidents on the Genaveh-Deylam road based on the missions of the Emergency Medical Center 

**Published:** 2019-08

**Authors:** Mahmud Rezai, Marjan Farvardin, Rezai Shafei

**Affiliations:** ^*a*^Emergency Medical Service Center, Local Health Center of Genaveh, Bushehr University of Medical Sciences, Bushehr, Iran.

## Abstract

**Background:**

The increasing expansion of the transportation industry and the rise of economic progress increases the use of extra-urban routes for this. Traffic accidents worldwide are known as one of the main causes of early death and death, and in Iran, the second cause of death and the first cause of the lost years is life. The conditions of work environment and climatic conditions in northern Provincial Bushehr province which connect to the provinces of Khuzestan, Fars, Kohgiluyeh and Boyerahmad, which are one of the most sought after axes in the outlying and international ways.

**Methods:**

This is a descriptive and analytical study whose data are based on the special form of missions carried out by Genaveh Medical Experts since the inauguration and change of the belt path in the Genaveh-Dilam route in 10 km of the city, from May 1998 to Avril 1998. Has been collected. These forms included mission time, location, injuries biographies, initial evaluation, prehospital triage and accident pattern. Statistical analysis was performed by researcher of this study using spss23 software.

**Results:**

In the limited time period, 13 emergency medical missions were performed. Most of the missions were related to Nowruz holidays. 23 were injured, 16 were male and 7 were women. The average age is 28 years. 100 drivers were male. 100% of the injured patients needed to be transferred to the treatment center. 48% trauma to the head, 43% trauma to the organs and 9% under supervision in the emergency department were monitored. 59% of the city medical center was sent to other cities of Bushehr province and neighboring provinces. In the investigation of the incident, most cases of car reversal occurred, only one case was reported after a motorcycle collision with a car. No death was reported.

**Conclusions:**

The results of this study showed that the incidence of accidents in this time efficiency is high in this path. It can be due to the high speed of young drivers, the lack of familiarity with the new changes on the outlet path of Genaveh -Dilam, or the presence of a turning back in the path that the road It has been suggested that, by equipping the road with safety equipment, reviewing the Genaveh-Deylam route and its standardization, it can be effective in reducing road accidents.

**Keywords:**

Accidents, Road safety, Emergencies

